# Excessive Food Buying in Saudi Arabia Amid COVID-19: Examining the Effects of Perceived Severity, Religiosity, Consumption Culture and Attitude toward Behavior

**DOI:** 10.3390/ijerph20043126

**Published:** 2023-02-10

**Authors:** Abu Elnasr E. Sobaih

**Affiliations:** 1 Management Department, College of Business Administration, King Faisal University, Al-Ahsaa 31982, Saudi Arabia; asobaih@kfu.edu.sa or abuelnasr@hotmail.co.uk; 2 Faculty of Tourism and Hotel Management, Helwan University, Cairo 12612, Egypt

**Keywords:** food security, COVID-19, perceived severity, food consumption culture, religiosity, personal attitude, food loss and waste, pandemic responses

## Abstract

The current study builds on both the Theory of Planned Behavior (TPB) and Protection Motivation Theory (PMT) to examine why consumers in Saudi Arabia engage in excessive food-buying behavior amid COVID-19. The study tests the direct impact of food consumption culture, perceived severity of COVID-19, and religiosity on excessive food-buying intentions and the indirect effect through attitudes toward excessive food buying. The results of the inner model using SmartPLS4 showed that the perceived severity of COVID-19 has a direct significant positive effect on attitudes toward excessive food buying and excessive food-buying intention. Despite food consumption culture being found to have no direct significant effect on excessive food-buying intention during the pandemic, it has a direct effect on attitudes toward excessive food buying. Surprisingly, religiosity was found to have a positive effect on consumers’ attitudes and excessive food-buying intentions. The results confirm that consumers misunderstood Islamic religious principles regarding food consumption, which does not accept excessive buying or food waste. Attitudes toward excessive food buying were found to mediate the relationship between food consumption culture, perceived severity of COVID-19, religiosity, and excessive food-buying intention. The results of the study are discussed and implications are highlighted for academics and policymakers.

## 1. Introduction

The Food and Agriculture Organization (FAO) has reported that one-third of the world’s food is wasted [1]. The situation in the Kingdom of Saudi Arabia (KSA) is even worse as KSA is among the top food-wasting countries in the world [2]. Surprisingly, food encompasses up to 50% of the overall waste in KSA, even more than paper, plastics, and other types of waste [3]. The international average of food waste per capita is 114 kg per year; however, this average is more than doubled in KSA to be 250 kg [4]. This food waste costs the KSA government 40 billion SR (Saudi Riyal) annually (about USD 11 billion) [5]. This is a challenge to the Saudi Vision 2030, food security, and sustainability in KSA [6]. This is especially true since KSA is not an ideal place for agricultural development due to limited arable land, tough weather, low rainfall, and water scarcity for agriculture [7]. Hence, KSA depends comprehensively on food imports to meet the needs of its people. Notwithstanding this, food waste in KSA is a major challenge [6]. Several causes were identified for this high rate of food waste in KSA, namely, economic prosperity, social events, and food consumption culture [6]. A recent study, amid the SARS-CoV-2 (COVID-19) pandemic, showed that excessive food-buying behavior is the major predictor of food waste in KSA [8]. The same study [8] also confirmed that excessive food buying is a direct response to the COVID-19 pandemic. Consumers were worried about virus transmission; hence, they engaged in excessive buying to limit their number of visits to food stores.

COVID-19 has affected the psychological and psychosocial state of humans around the world [9]. The severity of COVID-19 has increased fear and anxiety among communities worldwide, which pushed them to self-isolation [10]. Hence, this has encouraged consumers to practice excessive/unusual and/or panic buying behavior [10,11,12]. Recent research studies (see for instance, [8,12]) have shown that consumers in KSA, like many others around the world, engaged in excessive buying behavior, especially for essential items such as food and drink [12]. Despite the government of KSA trying its best to ensure the health, safety of its people, and availability of food stock, this was not enough to make Saudis desist from this excessive buying, which was increased amid the pandemic [12]. Nonetheless, this excessive buying behavior is an unhealthy practice and has a negative impact on individuals. Such behavior negatively affects the economy by increasing the prices since the shops might run out of stock [13]. It could lead to high food loss and waste [8]. Additionally, it becomes a threat to food security and sustainability in KSA [6].

Recent studies [8,12] on excessive buying behavior, especially in KSA, have shown that this behavior is not always a response to, or due to the impacts of, crises or pandemics; however, it is part of community cultures with economic prosperity. The study of Sobaih and Moustafa [12] showed that the COVID-19 pandemic has created unusual buying behavior among Saudis due to perceived severity, anxiety, and self-isolation. In other words, Saudis engaged in panic buying responding to the threat to their health because of the pandemic. Additionally, the study of Azazz and Elshaer [8] confirmed this assumption and found that social media has increased the effect of excessive food buying among Saudis during the pandemic, whereas religiosity has a negative association with this behavior. On the other hand, Baig et al. [6] found that this excessive behavior, which led to high food waste, is part of the Saudi culture supplemented with economic prosperity. Other studies, e.g., [14], showed that the excessive buying behavior could be a result of pleasure seeking. This confirms that excessive buying behavior is not always a natural response to crises, albeit it could be a result of various reasons as highlighted above.

The growing body of academic studies on excessive, panic, compulsive, or stockpiling buying behavior of essential needs, i.e., food and drinks, amid COVID-19 (see, for instance, [10,11,12]) has focused mainly on the direct impact of COVID-19 (e.g., perceived severity, anxiety, or self-isolation intention) on the spread of this behavior among consumers worldwide. Such research studies have highlighted the role of both academics and policymakers in managing such behavior to ensure food security and sustainability. On the other hand, there are limited studies, to the best of the researcher’s knowledge, which examine the impact of COVID-19 along with other factors such as consumption culture and religiosity, which were examined separately pre-COVID-19 [15]. The current study takes a novel attempt to examine the effect of COVID-19 alongside other factors, i.e., religiosity and consumption culture, on excessive food-buying behavior among consumers in KSA.

The study builds on both the Theory of Planned Behavior (TPB) [16] and the Protection Motivation Theory (PMT) [17]. The study draws on TPB to understand why consumers in KSA engage in excessive food-buying behavior amid COVID-19. The study adopts the TPB framework to understand the role of attitude toward behavior, as a major antecedent of excessive food-buying intention [18]. Attitudes toward behavior refers to a person’s positive or negative assessment of self-performance in relation to certain behaviors, which is excessive food buying in the current research. This study examines the role of attitude in the link between the perceived severity of COVID-19, food consumption culture, and religiosity and excessive food-buying intention. In addition, the PMT was adopted to explain how consumers respond with “coping appraisal” to the severity of COVID-19 “threat appraisal”. This study argues that consumers respond to the severity and threat of COVID-19 by their positive attitude and intention toward excessive food-buying behavior. The purpose of the current study is to test the direct effects of religiosity, food consumption culture, and perceived severity of COVID-19 on consumers’ excessive food-buying intention as well as the indirect effects via the consumers’ attitudes toward behavior. Understanding these relationships enables a better understanding of excessive buying behavior among consumers in KSA, which contributes to better management of food loss and waste that have become a threat to food security in KSA.

After the introduction of this study, the next section of this manuscript develops the hypotheses of the study including the direct relationship between the perceived severity of COVID-19, religiosity, and food consumption culture and excessive food-buying intention. It also reviews the indirect relationships through attitudes toward behavior. Section 2 also presents the theoretical model of the study. Following this, Section 3 shows the adopted methodology in the current study. This includes the instrument adopted, the study population and sample, procedures of data collection, and data analysis methods. Section 4 presents the results of the study (whether direct or indirect relationships) and the research structural model. Section 5 shows a discussion of the study’s results. Section 6 presents the implications of the study for academics as well as policymakers, especially in KSA. Section 7 concludes the study and highlights its major limitations.

## 2. Hypotheses Building

### 2.1. Food Consumption Culture, Attitudes toward Behavior, and Excessive Food Buying

According to Fourst [19] (p. 43), food culture is defined as “a distinct habits and consumer patterns in relation to food, which have established themselves over generations, such that they compose an entire tradition, which is often different from region to region.” Whereas excessive or impulsive buying refers to a type of buying behavior whereby individuals buy more than they used to [20]. Cultural norms have an impact on all aspects of people’s everyday lives; hence, an individual’s culture influences their practice toward food buying [21]. In that sense, Musaiger [22] indicated that food consumption culture is shaped by many variables, including social culture, which includes food pricing, income, food beliefs, special occasions, and food preferences.

In terms of Saudi culture, the Saudi people are known for their generosity, notably during their occasions such as the festivals of Eid, Ramadan month, Hajj season, weddings, and celebrations. Furthermore, the per capita income has expanded considerably in recent decades, owing mostly to oil wealth, while food prices are subsidized by the KSA government; these variables have resulted in a high degree of excessive food-buying behavior [7,15]. Earlier studies (e.g., [15,23,24]), emphasized that Saudis pay high attention to gracious hospitality and welcome to exceed their visitors’ expectations through introducing plentiful food. This kind of culture and traditions simply required buying food in bulk, especially at Eid, weddings, and parties, when lavish dishes are commonly served with lots of fresh and bountiful food. Likewise, Zayat [25] added that during Ramadan, Muslim people, i.e., Saudis, buy more food than they need. There is evidence that food consumerism culture has influenced the attitude and intention of Saudi people toward excessive buying. First, Baig et al. [7] asserted that the food consumption culture of Saudis significantly influences their attitude toward food buying because they have a good income and they have a high level of hospitality and generosity that increases their tendency and desire to buy food in bulk. Second, the subsidized price of food by the KSA government and their good income encourage Saudis people to buy food in an excessive way. Based on the above evidence considering the food consumption culture of Saudis, it can be hypothesized:

**Hypothesis 1 (H1).**  *Food consumption culture positively influences attitudes toward excessive food buying.*

**Hypothesis 2 (H2).**  *Food consumption culture positively influences excessive food-buying intention.*

### 2.2. Perceived Severity, Attitudes toward Behavior, and Excessive Food Buying

Perceived severity refers to the perception of the likelihood of unfavorable consequences when they take or refrain from a certain action [13]. Perceived severity, a sub-dimension of the health belief model, makes people feel compelled to engage in self-protective behavior in order to lower their risk perceptions as the PMT framework [17] implies. When exposed to environmental stress, such as stress resulting from the pandemic, people may be compelled to use a variety of coping mechanisms to handle the stress and preserve their health and well-being [13]. Earlier studies have indicated that perceived severity has been linked to excessive buying. To illustrate this, Sneath et al. [26] and Kennett-Hense et al. [27] asserted that in order to cope with unpleasant feelings such as worry and a diminished sense of security, people could spend more on purchasing in an excessive way. The worry of not finding food in the future because of uncertainty with the pandemic causes consumers to worry and carry out excessive buying [28,29]. Furthermore, a study conducted by Sobaih and Moustafa [12] confirmed that perceived severity significantly and positively affects the panic purchasing of Saudi people amid COVID-19. Panic buying is the purchase in bulk of a certain item or commodity because of immediate worries about a shortage or price increase [30,31]. Hence, we could argue that:

**Hypothesis 3 (H3).**  *Perceived severity positively influences attitudes toward excessive food buying.*

**Hypothesis 4 (H4).**  *Perceived severity positively influences excessive food-buying intention.*

### 2.3. Religiosity, Attitudes toward Behavior, and Excessive Food Buying

The Saudi Arabian people are categorized as a religious society, which shapes social behavior. The influence of religion on many aspects of consumers’ lifestyles eventually affects how they behave [6]. Forghani et al. [32] argued that religiosity has frequently been identified as a crucial variable that significantly affects consumer purchase decisions. Rakrachakarn et al. [33] stated the remarkable findings that religiosity has an impact on a variety of customer behaviors, which consequently changes how they behave while making purchases. Religious beliefs and morals have an impact on their decisions regarding accepting or rejecting particular items [34,35]. One important factor in determining how people behave is their adherence to God’s laws. This argument’s foundation is the idea that all heavenly religions, including Christianity and Islam, indicate that the environment was created by God, and that as a result, everyone in society should push others to preserve it and its resources, including food. Religion emphasizes the idea that everyone will face consequences for their behavior and that God listens, sees, and knows all they do. As a result, those who look at God as their primary source of guidance may worry about whether their acts are in accordance with his or her will. Additionally, religion directs people toward certain attitudes and behaviors [15,36].

The current research suggests that religiosity has a significant impact on buying behavior. A study finding by Minton et al. [37] reported that food consumption was correlated to rules and regulations of religion. This argument is twofold. Firstly, a person’s beliefs provide the foundation for their attitudes and behaviors. Secondly, it may be assumed that consumers who believe in God will be aware of, and accept the limits and laws put on, excessive buying in their everyday lives since frugality in shopping and food intake can be described as a devotion to God. To that end, a study conducted by Azazz and Elshaer [8] confirmed that religiosity has a negative relationship with unusual buying behavior among consumers, particularly in KSA. This is because it is forbidden by Allah for Muslims to engage in excessive buying behavior. Therefore, we propose the following:

**Hypothesis 5 (H5).**  *Religiosity negatively influences attitudes toward excessive food buying.*

**Hypothesis 6 (H6).**  *Religiosity negatively influences excessive food-buying intention.*

### 2.4. The Relationship between Attitude toward Behavior and Excessive Buying Intention

According to Ajzen [38], an individual attitude toward a behavior is defined as a positive or negative assessment of self-performance in relation to a certain behavior. It is a significant determinant of behavioral intention [39]. Commonly, behavioral intention rather than behavior has been connected to an attitude toward behavior [40]. The TPB explains the significance of attitudes toward behavior in shaping intention and, consequently, behaviors [40]. Referring to TPB, any social conduct may be explained by the behavior’s antecedents. Therefore, behavior is influenced by the intention to act. Consumers’ intention to engage in excessive purchasing can be linked to a dislike of the risk of scarcity and a heightened sense of financial security [41], resulting in consumers making illogical judgments at times [42]. The study of Elshaer et al. [15] confirmed a positive influence of attitude toward food waste on food waste intention among Saudis consumers. Hence, we assume that:

**Hypothesis 7 (H7).**  *Attitude positively influences excessive food-buying intention.*

### 2.5. The Mediating Effect of Attitude toward Behavior on the Link between Food Consumption Culture, Perceived Severity, Religiously, and Excessive Food-Buying Intention

**Figure 1:**
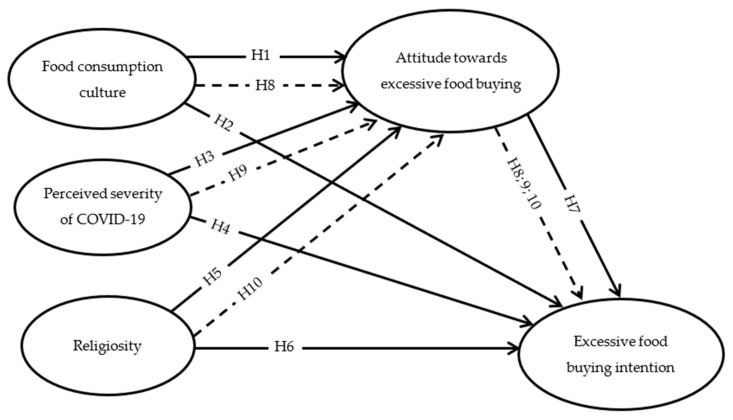
Effects of food consumption, perceived severity, religiosity, and attitude on excessive food-buying intention.

An attitude toward behavior is the evaluation of predicted behavioral outcomes expressed either positively or negatively [38]. The TPB provided a clear explanation of the significance of attitudes in determining intention and, ultimately, behavior [40]. In the context of buying behavior, the correlation between attitudes and intentions to shape consumers’ buying behavior has been validated by empirical investigations [41,42]. Religion is an important factor in determining the values that affect consumer attitudes. Martin and Bateman [43] discovered that individuals with high levels of religiosity also exhibited higher levels of environmental attitudes. Similarly, Ghazali et al. [44] found that religious beliefs have a favorable influence on environmental concerns, green buying attitudes, and green purchase intentions. The current study proposes that religiosity can influence attitudes toward excessive buying because it provides extrinsic benefits (e.g., rational money management), and avoiding profusion purchasing can be linked to intrinsic benefits (e.g., feeling satisfied due to their having obeyed God’s orders). Such benefits can trigger an overall positive inclination toward excessive buying. In that sense, a study conducted by Elshaer et. al. [15] confirmed that attitudes toward food waste mediate the relationship between religiosity, food consumption culture, and food waste intention among consumers in KSA. In the context of perceived severity, it was confirmed that severity has been linked to attitude toward excessive buying [10,11,12]. However, this study is a new attempt to test the mediating role of attitude in the link between the perceived severity of COVID-19 and excessive food-buying intention. Attitude toward excessive buying could mediate the relationship between religiosity, perceived severity, and food consumption culture and excessive buying intention (Figure 1). Consequently, we suggest:

**Hypothesis 8 (H8).**  *Attitude has a mediating effect on the relationship between consumption culture and intention toward excessive buying.*

**Hypothesis 9 (H9).**  *Attitude has a mediating effect on the relationship between perceived severity and intention toward excessive buying.*

**Hypothesis 10 (H10).**  *Attitude has a mediating effect on the relationship between religiosity and intention toward excessive buying.*

## 3. Methods

### 3.1. The Research Instrument

This research implemented a quantitative approach using a questionnaire survey with pre-tested research items. The questionnaire contained seven sections. Section 1 deals with the demographics of respondents, i.e., age, gender, and education. Section 2, Section 3 and Section 4 contain the independent variables, namely, food consumption culture (FCC), perceived severity (PS) of COVID-19, and religiosity (R). The scale items of these variables were taken from Aktas et al. [45], Omar et al. [10], and Minton et al. [46], respectively. Section 5 asks respondents about their attitude (A) toward excessive buying [38], whereas Section 6 includes the items about excessive food-buying (EFB) intention [47]. The items of Section 2, Section 3, Section 4, Section 5 and Section 6 are presented in Appendix A. Respondents were asked to state the level of agreement with each item on a scale ranging from 1 to 5 (1 = strongly agree and 5 = strongly disagree). The last section (Section 7) encourages respondents to comment about their food-buying behavior amid COVID-19.

### 3.2. Sampling and Data Collection Process

Due to the impact of COVID-19 and inability to access the respondents physically, the questionnaire survey was developed and distributed to online consumers in Saudi Arabia. The research implemented the recommendations of Evans and Mathur [48] for ensuring quality data collection using an online survey. This started with implementing a pre-tested research instrument as discussed in Section 3.1. The questionnaire was then reviewed by 20 professors of management. The questionnaire was developed online using Google Forms Platform and then reviewed by another 5 professors for face and content validity. The questionnaire started with an introduction welcoming the respondents and explaining the purpose of the research and then asking respondents for their consent to participate in the research. The introduction also confirmed the protection of respondents’ privacy. The questionnaire was distributed with support from a company that specialized in data collection. The link of the questionnaire was administered by a researcher and the company for collecting data representative of Saudi society. The link was sent to the respondents via emails and social media sites/groups. The data were collected over two months, i.e., January and February 2021. There was a regular check of the responses to ensure completeness.

The population of this research is the Saudi consumer since the researcher is based in Saudi Arabia. A sample of at least 384 responses was targeted. This sample size was suggested by Krejcie and Morgan [49] for a population of a million or above. A total of 810 complete responses were collected and valid for data analysis. This sample size was satisfactory according to Krejcie and Morgan [49]. It is also compared favorably with other similar studies [10,12].

### 3.3. Data Analysis Technique

This research adopted Smart partial least squares (SmartPLS) Version 4 software for data analysis. This software was adopted to examine the research hypotheses, whether direct or indirect relationships. SmartPLS is highly acknowledged as an appropriate tool for developing a structural model as undertaken in the current study. This research adopted the approach of Leguina [50] to examine the research conceptual model (Figure 1). The validity of the research outer measurement model was examined using the criteria proposed by Hair et al. [51] such as standardized factor loading (SFL) (>0.7); composite reliability (CR) (>0.7); average variance extracted (AVE) (>0.5); normed fit index (NFI) (>0.90); Standardized root mean square Residual (SRMR) (<0.08); R^2^ (>0.1); and Stone-Geisser Q2 (>0.0).

The research adopted several steps to address the likelihood of common method of variance (CMV) in the data since this research used a self-reporting online survey. First, as discussed in Section 3.1, respondents were assured of confidentiality. Second, the dependent variable of excessive food buying was placed before the independent variables. Third, the research adopted Harman’s single-factor technique. This involves underrating exploratory factor analysis for all factors using SPSS with only one factor retrieved without rotation. The finding confirmed that CMV is not an issue as one variable explained 35% of the variance [51].

## 4. Results

### 4.1. Respondets’ Demographics

Respondents were almost of equal gender participation, where males were 52% and females 48%. The age of all participants was above 18 years old. The majority of them (41%) were between 31 and 40 years old, followed by those between 41 and 50 years old (33%), then above 50 years old (18%), and less than years old but over 18 (8%). The mainstream respondents (62%) held a bachelor’s degree or equivalent. This was followed by those holding a diploma (two-year degree after high school) (19%), then those with a postgraduate degree (12%), and finally respondents holding a high school or equivalent degree (7%).

### 4.2. The Measurement Model

As a part of examining the research model, it is important to assess the validity and reliability of the measurement model. This research adopted the guidelines provided in literature reviews [50,51]. Hence, the research assessed discriminant and convergent validity. This includes assessing Cronbach’s alpha (*a*), composite reliability (CR), and average variance extracted (AVE) (see Table 1). Table 1 shows all measures confirm acceptable alpha values ranging between 0.703 and 0.937, which were all above 0.7. Additionally, the values of CR were also above 0.70. The values of alpha and CR in Table 1 confirm that the instrument has adequate internal reliability. Furthermore, the value of AVE was above 0.5 for all the variables confirmed. The standardized factor loading of all items was above 0.7, giving more support to the convergent validity [50].

This research adopted the approach of Leguina [50] to assess the discriminant validity by assuring three measures: factor cross loading, the Fornell–Larcker criterion, and heterotrait/monotrait ratio (HTMT). As Table 2 shows, the standardized factor “outer-loading” of each latent reflective variable is considerably more than the cross loading with other items.

As Leguina [50] suggested, the second criterion for ensuring the discriminant validity after checking outer-loading and cross-loadings is to check the diagonal values of AVE (in bold in Table 3). This has to be greater than the inter-variable correlation coefficient, as Table 3 shows. Additionally, the HTMT values should be less than 0.90 to confirm discriminant validity, which is the case in Table 3.

### 4.3. The Research Structural Model

Following the confirmation of the validity and reliability of the scale, as well as the outer model, the next step is to assess the structural model. A structural equation model was conducted to test the hypotheses of the research. In the beginning, it is important to ensure the goodness-of-fit indices of the model [50,51,52,53]. The indices (Table 4) confirm that the structural model has excellent fit for the data and has good predictive power. All the indices were acceptable compared to threshold values suggested by Hair et al. [51]. Based on these results, the study can undertake the structural analysis.

This research adopted a bootstrapping approach using SmartPLS 4 to assess the direct and indirect relationships. There are seven direct research hypotheses and three mediating ones. Table 5 shows that the effect of food consumption culture on attitude toward excessive food-buying intention is positive and significant (β = 0.766, *t* = 17.318, *p* < 0.001), supporting Hypothesis 1. However, the direct effect of food consumption culture on excessive food-buying intention is not significant, which rejects Hypothesis 2. The effect of the perceived severity of COVID-19 on both attitude toward excessive food buying (β = 0.130, *t* = 2.971, *p* < 0.01) and excessive food-buying intention (β = 0.208, *t* = 4.450, *p* < 0.001) is positive and significant, confirming Hypotheses 3 and 4, individually. Surprisingly, the effect of religiosity on attitude toward excessive food buying (β = 0.117, *t* = 2.807, *p* < 0.01) and excessive food-buying intention (β = 0.143, *t* = 2.245, *p* < 0.05) is positive and significant, which rejects Hypotheses 5 and 6, individually. Additionally, the effect of attitude toward excessive food-buying intention is positive and significant (β = 0.425, *t* = 4.686, *p* < 0.001), confirming Hypotheses 7 (Table 5).

**Figure 2:**
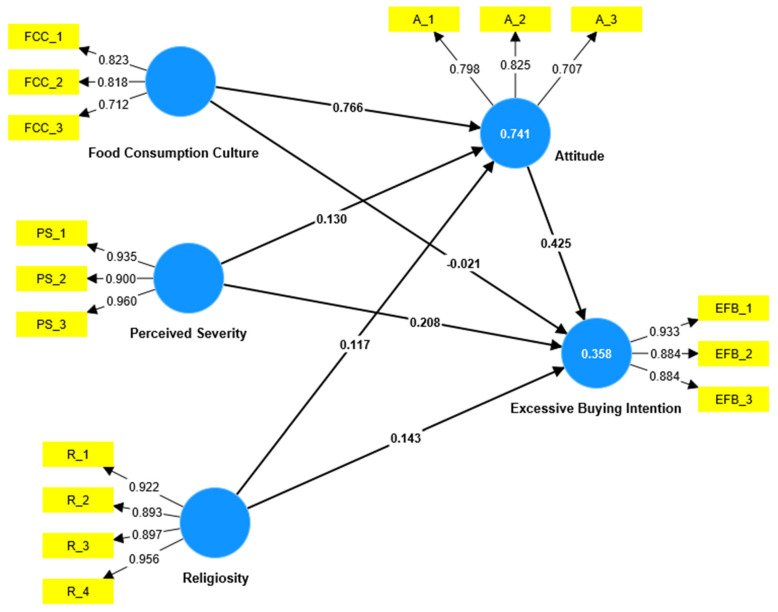
The research inner model.

For examining the mediation factors’ specific indirect effect in the bootstrapping report, SmartPls 4 was employed. As Table 5 shows, attitude toward excessive food buying mediates the relationship between food consumption culture and excessive food-buying intention (β = 0.326, *t* = 4.432, *p* < 0.001), confirming Hypothesis 8. Furthermore, attitude toward excessive food buying mediates the relationship between the perceived severity of COVID-19 and excessive food-buying intention (β = 0.055, *t* = 2.941, *p* < 0.01), supporting Hypothesis 9. Finally, attitude toward excessive food buying mediates the relationship between religiosity and excessive food-buying intention (β = 0.050, *t* = 2.430, *p* < 0.05), supporting Hypothesis 8 (see Figure 2).

## 5. Discussion

The current study is a response to the excessive food buying among consumers in KSA, which has become the norm amid the COVID-19 pandemic. The current study draws on the Theory of Planned Behavior (TPB) [16] and the Protection Motivation Theory (PMT) [17] to examine why consumers in KSA engage in excessive food-buying behavior amid COVID-19. The TPB framework was adopted to assess the role of attitude toward behavior, as a major antecedent of excessive food-buying intention. More specifically, the study examines the role of attitude in the link between the perceived severity of COVID-19, food consumption culture, and religiosity and excessive food-buying intention. Moreover, the study adopted the PMT to understand consumers’ coping mechanisms for the health threat of COVID-19.

The results of SmartPLS4 showed that the effect of food consumption culture on attitude toward excessive food-buying intention is positive and significant. This supports the assumption that Saudi culture serves plentiful food, even more than can be eaten, to express their hospitality [15,23,24]; this was clearly translated into their attitude toward excessive buying. Baig et al. [7] supported this notion that the food consumption culture of Saudis significantly influences their attitude toward excessive food buying. This is because they have a good income and they have a high level of hospitality and generosity that increases their tendency and desire to buy food in bulk. However, the direct effect of food consumption culture on excessive food-buying intention was not significant. This could be because consumers during the pandemic are self-isolated and there were no social meetings due to the health threat. Thus, the food consumption culture among Saudis during the pandemic failed to promote excessive food-buying intention.

On the other hand, the effect of the perceived severity of COVID-19 was positive and significant on both attitude toward excessive food buying and excessive food-buying intention. This finding is in line with the PMT framework [17], whereby consumers in KSA, like in many other countries around the world, tried to cope with the perceived severity of COVID-19 through their positive attitude and intention of excessive food buying. The results of the current research support previous studies that people engage in unusual or panic buying as a coping appraisal to the threat perceived because of the COVID-19 pandemic [10,12]. Amid COVID-19, consumers in KSA were found to have a positive attitude and intention of excessive food buying due to their perception of COVID-19’s severity as being life-threatening. Their worries and uncertainty about the pandemic enhanced this intention of excessive food buying [10,11,12]. Our research confirms the results of recent research [54,55] on the influence of COVID-19 on increasing food loss and waste behavior among consumers in various countries. However, recent research [56,57] has argued that this time of pandemic is an opportunity to raise awareness among consumers about food loss and waste.

One of the unexpected results was the effect of religiosity on attitude toward excessive food buying and excessive food-buying intention, which was found to be positive and significant. Unlike the results of previous studies about the negative effect of religiosity on excessive buying amid COVID-19 [8], the current study showed a positive influence on both attitude and intention of excessive food buying. These positive attitudes and intentions among respondents confirm a misunderstanding of the Islamic religion (since all Saudis are classified as Muslims). The Islamic religion clearly prohibits excessive food consumption, as stated in the Quran (the Holy book of Muslims) and Hadith (the messages of Messenger Prophet Mohammed). However, they misinterpret part of the texts. For example, “*And they give food in spite of love for it to the needy, the orphan, and the captive*” (Verse 8, Al-Insān) and “*Then he went to his family and came with a fat [roasted] calf”* (Verse 26, Adh-Dhāriyāt). These verses in the Quran quoted by the respondents in Section 7 of the questionnaire are just some examples that confirm a misunderstanding of some Islamic principles. Some respondents used these verses to argue that their Islamic religion confirms hospitality. However, these verses have a specific context and cannot be considered as a justification for excessive food consumption. Certainly, Islam does respect hospitality but, at the same time, confirms no excessive or food waste, as it is mentioned in the Quran that “*…. and eat and drink, but be not excessive. Indeed, He likes not those who commit excess*” (Verse 8, Al-A‘rāf).

The results of the current research support the TBP framework that attitude toward behavior is an antecedent of behavioral intention [38,39]. Hence, the results confirmed a positive and significant effect of attitude toward excessive food buying on excessive food-buying intention. This finding is in agreement with previous research [15] that the Saudi consumer’s attitude toward food waste was a predictor of food waste intention. Additionally, the results showed that attitude toward excessive food buying mediates the relationship between food consumption culture, perceived severity of COVID-19, religiosity, and excessive food-buying intention. Despite the results confirming no significant direct effect of food consumption culture on excessive food-buying intention, attitudes toward excessive buying mediated this relationship. Hence, the indirect effect was confirmed through attitude toward excessive buying.

## 6. Implications of the Study

The above results have several implications for academics. First, the results confirmed that excessive food buying amid COVID-19 is not just a direct response or a coping behavior to the health threat of the pandemic as PMT suggested, as other factors led to this behavior, such as food consumption culture and religiosity, whether directly or indirectly. It is important to note that Saudis engaged in excessive food buying in response to the health threat coming from the quick transmission of the virus. Despite the efforts of the Saudi government to provide free vaccinations, ensure social distancing, and provide different forms of support to the community [10], these efforts did not stop Saudis from engaging in excessive food buying. Second, the results extend the TBP framework that there are other antecedents of behavioral intention. The study showed that food consumption culture, perceived severity, and religiosity are all antecedents of excessive food-buying intention, directly or indirectly. Third, the study confirmed, for the first time, the mediating effect of attitude toward behavior on the relationship between food consumption culture, perceived severity, and religiosity and excessive food-buying intention. Attitude toward behavior was able to change the direct effect of food consumption culture on excessive food-buying intention, from insignificant to significant, through consumers’ personal attitudes. Hence, the current research contributed to both PMT and TPB frameworks.

The results also have several implications for decision makers. First, media campaigns are urgently needed to explain the correct Islamic principles about excessive food and the negative consequences of engaging in such behavior from an Islamic perspective. It is important that the different media campaigns consider the reduction in worries and stress of the community regarding the threat of the pandemic/crises in order to reduce their excessive food-buying behavior, which is often a result of this threat. Second, the role of social media cannot be underestimated since it encourages this excessive behavior [8]. However, it has been used as a powerful tool for sending important messages about public health, as well as the assurance of the value of food saving and food security and sustainability. Third, Muslim leaders can play an important role in this by showing a negative attitude toward excessive food through different media channels. Fourth, managing this excessive food-buying behavior will contribute to food security and the achievement of the United Nations Sustainable Development Goals (SDGs), especially goals number 1 and 2, relating to no poverty and zero hunger, respectively.

## 7. Conclusions 

The current study is an attempt to understand why the Saudi consumer engages in excessive food buying, especially amid COVID-19. The results showed that the perceived severity of COVID-19 has a direct significant positive effect on attitudes toward excessive food buying and excessive food-buying intention. Food consumption culture was found to have no direct significant effect on excessive food-buying intention, albeit it has a direct effect on attitudes toward excessive food buying. It was surprising that religiosity has a positive effect on both consumers’ attitudes and excessive food-buying intention. This implies a misunderstanding of Islamic principles, which led to the positive effect of religiosity on attitudes toward excessive food buying. Additionally, attitude toward excessive food buying mediates the relationship between food consumption culture, perceived severity of COVID-19, religiosity, and excessive food-buying intention. This means that attitudes toward excessive food buying have the ability to change the direct relationships, which was the case for the effect of food consumption culture on excessive food-buying intention.

The study adopted an online survey due to the effect of COVID-19; hence, respondents may not be representative of the Saudi population. Additionally, the study did not examine the effect of respondents’ demographics, e.g., gender, age, and education, on the study results, which could be an opportunity for further research. One of the important variables that could be examined in future studies is the moderating effect of respondents’ income on the examined relationships in the current study. It will be interesting to examine the role of economic prosperity on excessive food-buying behavior.

## Figures and Tables

**Table 1:** Results of measurement model.

Dimensions/Variables	Loadings	*a* Value	C.R	AVE
Attitude		0.703	0.701	0.605
A_1	0.798			
A_2	0.825			
A_3	0.707			
Excessive Buying Intention		0.883	0.889	0.811
EFB_2	0.933			
EFB_3	0.884			
EFB_2	0.884			
Food Consumption Culture		0.702	0.708	0.618
FCC_1	0.823			
FCC_2	0.818			
FCC_3	0.712			
Perceived Severity		0.924	0.937	0.869
PS_1	0.935			
PS_2	0.900			
PS_3	0.960			
Religiosity		0.937	0.938	0.842
R_1	0.922			
R_2	0.893			
R_3	0.897			
R_4	0.956			

**Table 2:** The outer-loading and cross-loadings.

	Attitude	Excessive Buying Intention	Food Consumption Culture	Perceived Severity	Religiosity
A_1	**0.798**	0.486	0.684	0.349	0.436
A_2	**0.825**	0.396	0.690	0.238	0.263
A_3	**0.707**	0.379	0.583	0.288	0.231
EFB_1	0.546	**0.933**	0.474	0.409	0.292
EFB_2	0.478	**0.884**	0.376	0.349	0.261
EFB_3	0.440	**0.884**	0.335	0.323	0.442
FCC_1	0.714	0.401	**0.823**	0.288	0.309
FCC_2	0.656	0.317	**0.818**	0.187	0.224
FCC_3	0.607	0.316	**0.712**	0.174	0.243
PS_1	0.393	0.402	0.240	**0.935**	0.362
PS_2	0.300	0.331	0.250	**0.900**	0.150
PS_3	0.351	0.384	0.293	**0.960**	0.237
R_1	0.388	0.298	0.350	0.189	**0.922**
R_2	0.340	0.348	0.299	0.132	**0.893**
R_3	0.396	0.343	0.301	0.354	**0.897**
R_4	0.367	0.354	0.268	0.327	**0.956**

**Table 3:** Fornell–Larcker criterion and Heterotrait/monotrait ratio (HTMT)—Matrix.

	Fornell–Larcker Criterion					Heterotrait/Monotrait Ratio (HTMT)—Matrix				
	1	2	3	4	5	1	2	3	4	5
1-Attitude	**0.778**									
2-Excessive Buying Intention	0.544	**0.901**				0.699				
3-Food Consumption Culture	0.541	0.442	**0.786**			0.232	0.560			
4-Perceived Severity	0.377	0.402	0.280	**0.932**		0.472	0.440	0.346		
5-Religiosity	0.407	0.367	0.331	0.276	**0.917**	0.502	0.405	0.411	0.285	

**Table 4:** Goodness-of-fit index.

Endogenous Variables	(R2)	(Q2)
Attitude	0.741	0.722
Excessive Buying Intention	0.358	0.286
Model Fit	SRMR	NFI
	0.044	0.902

**Table 5:** The results of hypotheses.

	Relationships	Beta (β)	(*t*-Value)	*p* Values	Results
H1	Food Consumption Culture -> Attitude	0.766	17.318	0.000	Accepted
H2	Food Consumption Culture -> Excessive Buying Intention	−0.021	0.242	0.809	Not Accepted
H3	Perceived Severity -> Attitude	0.130	2.971	0.003	Accepted
H4	Perceived Severity -> Excessive Buying Intention	0.208	4.450	0.000	Accepted
H5	Religiosity -> Attitude	0.117	2.807	0.005	Not Accepted
H6	Religiosity -> Excessive Buying Intention	0.143	2.245	0.025	Not Accepted
H7	Attitude -> Excessive Buying Intention	0.425	4.686	0.000	Accepted
H8	Food Consumption Culture -> Attitude -> Excessive Buying Intention	0.326	4.432	0.000	Accepted
H9	Perceived Severity -> Attitude -> Excessive Buying Intention	0.055	2.941	0.003	Accepted
H10	Religiosity -> Attitude -> Excessive Buying Intention	0.050	2.430	0.015	Accepted

## Data Availability

Data are available upon request from researchers who meet the eligibility criteria. The data can be requested from the author by email.

## Appendix A

**Table A1:** The research instrument.

Religiosity		Reference
R_1	I believe in God	Minton et al. [46]
R_2	I have no doubts that God lives and is real	
R_3	There is life after death	
R_4	The scripture for my religious affiliation is the word of God	
Food consumption culture		Aktas et al. [45]
FCC_1	It is my culture to serve a lot of food to show my hospitality	
FCC_2	I have a tendency to buy a few more food products than I need at the restaurant	
FCC_3	I serve more food than can be eaten to show my hospitality	
Excessive food-buying intention		Neff et al. [47]
EFB_1	I intend to purchase larger quantities of food than we currently require, when there are quantity discounts,	
EFB_2	I intend to purchase food items that we already had at home	
EFB_3	I intend to purchase items that were not on our shopping list	
Perceived Severity		Omar et al. [10]
PS_1	COVID-19 pandemic is a serious threat	
PS_2	COVID-19 pandemic is critical	
PS_3	COVID-19 pandemic is a life-threatening	
Attitude toward behavior		Ajzen [38]
A_1	I feel good when I buy more food than needed	
A_2	I was raised to believe that I should have more food at home	
A_3	I think I should buy as much food as I can	
